# Continuous Flow Synthesis of Anticancer Drugs

**DOI:** 10.3390/molecules26226992

**Published:** 2021-11-19

**Authors:** Mara Di Filippo, Marcus Baumann

**Affiliations:** School of Chemistry, University College Dublin, Science Centre South, Belfield, D04 N2E2 Dublin, Ireland; mara.difilippo@ucdconnect.ie

**Keywords:** flow synthesis, API, anticancer drug, process control, reaction telescoping, drug synthesis, continuous process, industrial application

## Abstract

Continuous flow chemistry is by now an established and valued synthesis technology regularly exploited in academic and industrial laboratories to bring about the improved preparation of a variety of molecular structures. Benefits such as better heat and mass transfer, improved process control and safety, a small equipment footprint, as well as the ability to integrate in-line analysis and purification tools into telescoped sequences are often cited when comparing flow to analogous batch processes. In this short review, the latest developments regarding the exploitation of continuous flow protocols towards the synthesis of anticancer drugs are evaluated. Our efforts focus predominately on the period of 2016–2021 and highlight key case studies where either the final active pharmaceutical ingredient (API) or its building blocks were produced continuously. It is hoped that this manuscript will serve as a useful synopsis showcasing the impact of continuous flow chemistry towards the generation of important anticancer drugs.

## 1. Introduction

Drug shortages remain a significant public health issue in the 21st century. All types of drugs are affected by this problem such as anticancer medicines, antimicrobial drugs, analgesics, opioids, cardiovascular drugs, radiopharmaceuticals, and parenteral products. This global problem furthermore has severe economic implications and affects society as a whole [1]. In the context of anticancer medicines, shortages are even more critical due to the precarious patient situation, which often cannot afford a delay in treatment or a replacement with an alternative drug due to adverse effects such as incompatibility with other medications or higher cost [2,3]. The shortage of a single medicine can have repercussions on many patient cohorts as the same product may be used to treat several conditions. It is therefore of utmost importance to avoid shortages of medicines to ensure patients have the highest survival rate possible.

**Figure 1:**
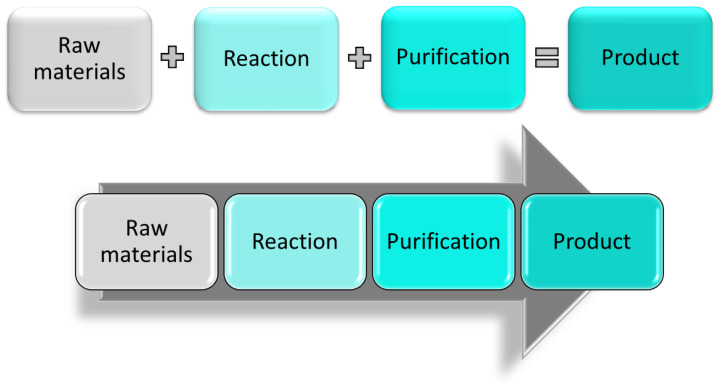
Stepwise batch manufacturing (**top**) and continuous manufacturing (**bottom**) representation.

Recently, both FDA (Food and Drug Administration) and EMA (European Medicines Agency) published updated reports analyzing this situation [4,5]. Drug shortages can occur for many reasons such as manufacturing and shipping problems or price changes and discontinuations of raw materials. The ongoing global pandemic has seen additional supply issues for several drugs due to the disruption of production and distribution networks [6]. Limited availability of drugs and their building blocks can also result from their production being suspended or disrupted due to contamination of the active pharmaceutical ingredient (API) during the manufacturing process, manufacturing capacity issues, or simply due to the inability to produce as much product as required. Manufacturing issues occur because most pharmaceutical companies strongly rely on traditional batch processing and related supply networks. In this type of discontinuous processing, the raw material is processed in large vessels, in which the chemical reaction is allowed to proceed for a given period of time before the product is discharged and eventually purified (Figure 1). If a problem occurs during this process, all the materials used are compromised and must be discarded, losing a significant amount of chemicals, money, and production time, especially if the issue occurs at the later stages of synthesis. Even when the production process runs smoothly, most of the time the crude product needs to be purified thus rendering batch processes time consuming, labor-intense, and environmentally unfavorable.

To improve the manufacturing process, the chemical industry has started to embrace more advantageous emerging technologies, particularly continuous flow chemistry which allows for various improvements on the manufacturing process as documented previously [7,8,9,10,11,12,13]. In the last 20 years, a growing number of publications have demonstrated the positive impact of continuous manufacturing in industrial applications and now, continuous flow processing is recognized as a game-changer that pharmaceutical companies have largely welcomed [14,15,16,17,18].

Compared to batch manufacturing, continuous manufacturing offers higher quality products and less batch-to-batch variability because of the high control over reaction conditions (e.g., temperature, pressure, and reaction time). For the same reason, flow technology enables chemists to easily perform reactions that would be very challenging in batch mode [19] due to extreme conditions, such as high- and low-temperature conditions [20,21,22,23], high pressure [24,25,26], the presence of highly reactive and unstable intermediates [27], as well as photo- or electrochemical processing at scale [28,29,30,31,32]. The modular nature of this technology and the robustness of individual reactor components not only provide flexibility but also facilitate the expansion of the applications of flow reactors to different industrial processes, which can mitigate production-chain incidents [7]. Additionally, the closed environment of flow reactor systems provides safer working conditions, preventing the operator from being in direct contact with hazardous chemicals [33,34]. The small equipment requires less laboratory space and reactor miniaturization intrinsically improves the quality of the reactions due to the excellent mass and heat transfer. Continuous flow processes can be telescoped and automated [35,36] aided by the integration of suitable process analytical technologies (PAT) and purification modules, which accelerate the production retaining product quality and increase product throughput [37,38]. Telescoped processes also improve the green aspects of the manufactory process because the product of a reaction does not need to be isolated and stored before being used in the following step but can directly flow to the next reactor [33,39,40,41].

**Figure 2:**
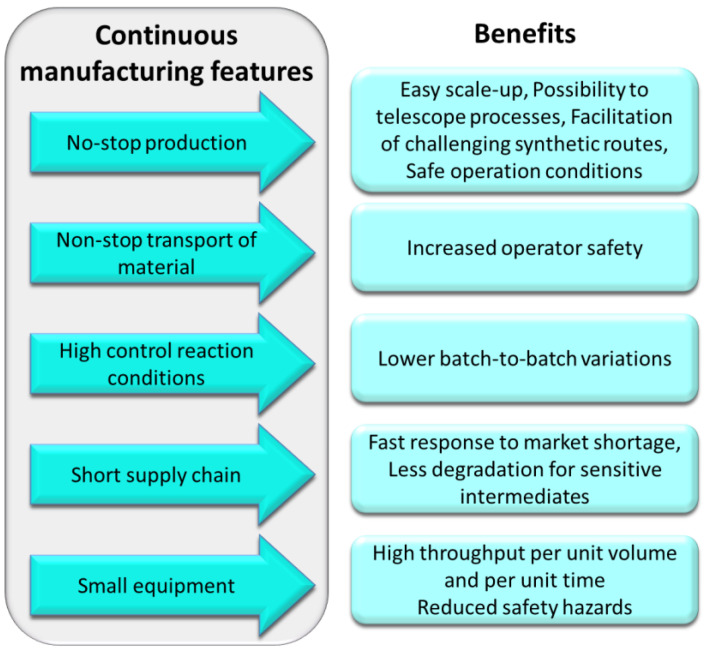
Summary of advantages of flow chemistry relevant to industrial applications.

Because of the many advantages (Figure 2) that can be leveraged by developing continuous flow processes over batch routes, many pharmaceutical companies have been investing heavily into this technology to produce fine chemicals, as well as drugs and their precursors. In this review, we wish to highlight the use of flow processes applied to the synthesis of important anticancer drugs and their building blocks as reported within the last five years (2016–2021).

## 2. Discussion

The following Table 1 lists the target molecules discussed in the proceeding sections of the review along with the predominant tumor targets.

## 3. Syntheses of Anticancer Drugs in Flow Mode

### 3.1. Lomustine

Lomustine (**4**) is a nitroso urea species widely used in anticancer therapies, especially to treat CNS tumors, throat and larynx tumors, lymphogranulomatosis, Hodgkin’s lymphoma, lung, and gastrointestinal tract tumors. This drug acts as a DNA-alkylating agent that produces chloroethyl carbenium ions and carbamylated intermediates in vivo [42,43,44]. The main cytotoxic effect of these species results from their ability to form an adduct via the oxygen atom of guanine causing DNA cross-linking. This adduct interferes with key cellular processes such as DNA replication leading to cell death via apoptosis [45,46].

Lomustine, which is sold under the brand name Gleostine^®^, is used orally for treatment every six weeks. Recently, in part due to the new regulatory challenges of handling unstable compounds of this type, the price of Gleostine^®^ has increased considerably [47]. The possibility of making this drug via a more convenient on-demand methodology may contribute to reducing its cost in the future.

Thompson et al. published an interesting method for the synthesis of this anticancer compound in continuous flow mode [48]. This involves a telescoped two-step sequence without isolation of the intermediate. The use of desorption electrospray ionization mass spectrometry (DESI-MS) as an in-line analysis technique was beneficial in the optimization process to evaluate the impact of solvent, concentration, and nitrosation reagent choice on the efficiency of the flow process. 

**Scheme 1:**
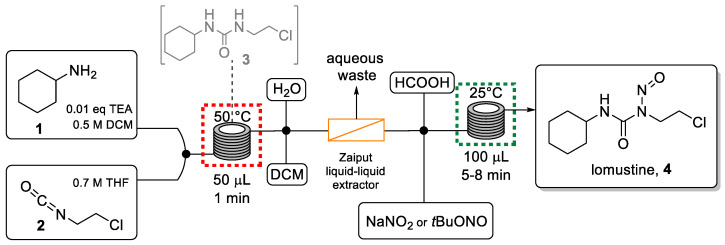
Telescoped flow synthesis of lomustine.

The telescoped approach (Scheme 1) was performed using two microreactors (0.5 mL and 1 mL volume) made from fluorinated ethylene propylene (FEP) tubing. The first carbamylation reaction involved combining both reagent solutions via a microreactor maintained at 50 °C and was achieved with a residence time of only one minute.

The exiting solution was then diluted with a mixed solvent system (H_2_O/DCM) to prevent reactor clogging due to the low solubility of urea intermediate **3**. The resulting stream was directed into a Zaiput liquid-liquid separator to remove the water-soluble base (TEA) from the system and avoid the consumption of the nitrosation agent in the second step. The organic solution was combined with a solution of the nitrosation reagent (NaNO_2_ or *t*BuONO) and formic acid before entering a second microreactor to perform the nitrosation process. With this method, lomustine was generated in a yield of 63% in a residence time of only 9 min. This equaled a productivity of 110 mg/h of **4**, equivalent to one dose every 2 h. This result clearly shows the attractive features of the flow process compared to the analogous batch route which was slower and generated a lower yield. [49] After a simple purification procedure (extraction, filtration, and washing), lomustine was obtained with a purity comparable to the commercially available drug. 

This methodology shows the potential for reducing production costs by using simple reactor setups and inexpensive reagents. The possibility to integrate an intermediate work-up into the continuous process furthermore allows for faster routes. More importantly, the closed system provided by the flow reactor technology means that operators are not in contact with potentially harmful alkylating agents, such as 1-chloro-2-isocyanatoethane (**2**) and cyclohexylurea intermediate **3**, enhancing the safety aspect of the procedure.

Recent efforts by Diab et al. have furthermore investigated the reaction kinetics for the continuous synthesis of lomustine which adds further value to these efforts [50].

### 3.2. Tamoxifen

Tamoxifen (**12**) is one of the most widely prescribed estrogen receptor modulators used to treat breast cancer, however, it also finds use in the treatment of infertility, gynecomastia, and other disorders [51,52,53,54]. The typical daily dose is 10 mg, with a treatment lasting over 5 years. Since there is a significant demand for this drug, it is vitally important to provide an efficient synthesis.

**Scheme 2:**
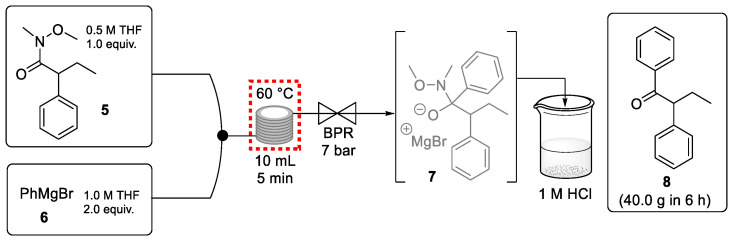
Continuous-flow preparation of ketone **8** from Weinreb amide **5**.

Ley et al. reported a continuous-flow process to generate (E/Z)-tamoxifen with excellent outcomes [55]. An initial flow reaction concerned the preparation of the ketone starting material (**8**) through the addition of PhMgBr to Weinreb amide **5** (Scheme 2). This continuous process step provided 40 g of this ketone product in an excellent yield (97%) in only 6 h. Although the purity of the exiting stream was very high (>98%) an off-line acid quench was required to break down the metallated tetrahedral intermediate **7**. Therefore, the ketone preparation was not telescoped with the rest of the synthesis. It is important to note that the corrosive Grignard reagent was used safely at an elevated temperature using a back pressure regulator.

**Scheme 3:**
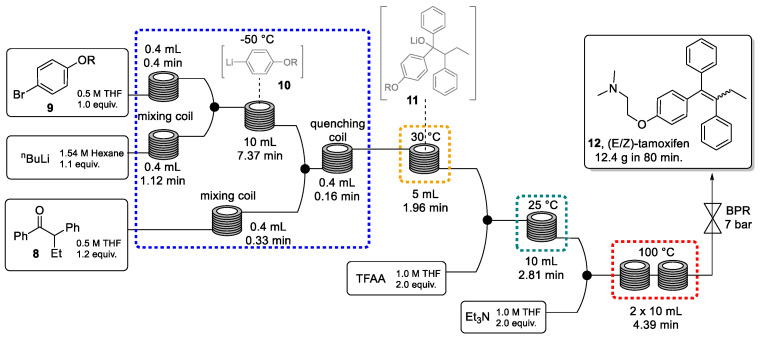
Telescoped synthesis of (E/Z)-tamoxifen through a continuous process.

Next, four sequential transformations in a continuous-flow system were envisaged to generate (E/Z)-tamoxifen (Scheme 3). This sequence utilized seven perfluoroalkoxy (PFA) reactor coils carefully integrated to perform the four key steps of the synthesis: (a) lithiation of aryl bromide **9** in a cryogenic reactor at −50 °C, (b) addition of the resulting aryl lithium species (**10**) to ketone **8** at 30 °C, (c) activation of the lithium alkoxide intermediate (**11**) using trifluoroacetic anhydride (TFAA) at room temperature, and (d) base-mediated elimination at 100 °C. The procedure generates tamoxifen (**12**) with an excellent productivity of 9.35 g/h, which is equivalent to one daily dose every 5 s. It is worth noting that batch processing would not provide the flexibility to easily vary temperatures as exploited in this continuous route. In addition, the continuous system provides fine control over reaction conditions minimizing side-product formation and at the same time maximizing productivity and product quality.

### 3.3. Imatinib

Imatinib (**18**) is the active API of the commercial anticancer drug Gleevec^®^. It is used for the treatment of chronic myelogenous leukemia and gastrointestinal stromal tumors [56,57,58]. The batch production relies on a multi-step sequence which is labor-intensive and time-consuming [59].

Jamison and co-workers demonstrated an effective strategy for the synthesis of this drug using readily available building blocks in a continuous fashion [60]. Inspired by the work published by the Ley group in 2010 [61], their approach foresees three strategic steps: (a) hydration of a nitrile, (b) chemoselective amidation, and (c) C-N cross coupling. 

The three synthetic steps were integrated into a single flow process that does not require in-line purification, solvent switching, or packed-bed apparatus, which is different from the work reported by the Ley group. 

**Scheme 4:**
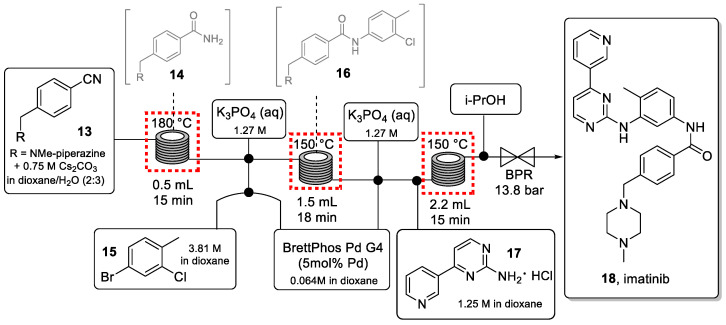
Continuous-flow synthesis of imatinib (**18**).

The telescoped process for the synthesis of imatinib is shown in Scheme 4.

**Figure 3:**
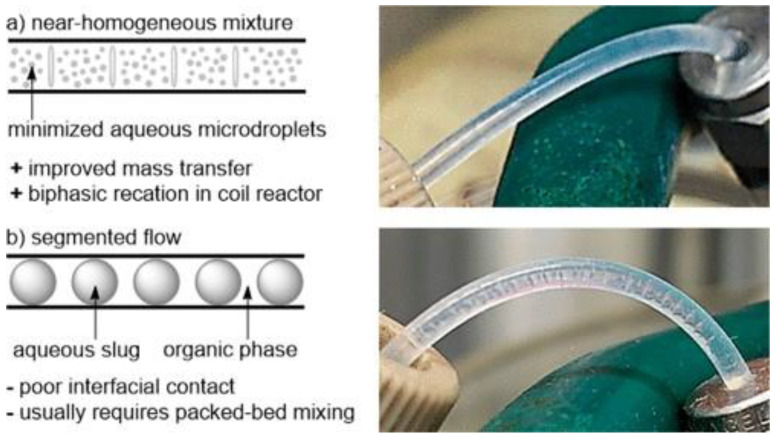
(**a**) Near-homogeneous mixture produced by a cross mixer with small diameter; (**b**) typical segmented flow of a biphasic mixture. Reprinted with permission from *Org. Lett.* 2019, *21*, 6112–6116. Copyright 2019 American Chemical Society.

In the first stainless-steel reactor coil, nitrile starting material **13** was quickly converted to the corresponding amide intermediate **14** via a hydration reaction using Cs_2_CO_3_. This high-temperature process generated a clean amide intermediate thus mitigating the need for purification. Prior to entering the second coil, the reaction stream was combined with a solution of aryl halide (**15**) and a Pd-precatalyst, as well as an aqueous solution of K_3_PO_4_. The three immiscible streams were mixed in a cross mixer with a small inner diameter (0.02″ id), which created a near-homogeneous mixture (Figure 3) capable of improving the interfacial contact between the organic and the aqueous phase.

Intermediate **16** was then combined with the amino-pyrimidine **17** and the previously employed Pd-catalyst to carry out the final C-N cross coupling in the last high-temperature reactor coil. Careful optimization of concentration and solvent system was effective to avoid the precipitation of salts that could otherwise lead to reactor blockages. 

Imatinib (**18**) was isolated in 58% yield with a total residence time of 48 min, producing 327 mg/h of this drug which is equivalent to almost 4 g of API in 12 h. The same methodology was used to synthesize imatinib analogs. Critical to the success of this telescoped flow approach were the optimization of individual flow rates, reagent stoichiometries as well as the concentrations of each stream to ensure high reactivity without problems due to precipitation and reactor fouling. In addition, using a backpressure regulator (BPR, 13.8 bar) enabled superheating the dioxane-based solvent system thus accelerating reaction kinetics and increasing the yield of the process. 

### 3.4. HSN608

HSN608 is a novel and potent inhibitor of the *Fms*-like tyrosine kinase 3 (FLT-3), a protein overexpressed in patients with acute myeloid leukemia (AML) [62,63].

The reported batch synthesis has several drawbacks such as high catalyst loadings, low yields, long reaction times, and the use of a potentially explosive coupling reagent (HATU) which would be a concern in a large-scale batch synthesis [64,65].

**Scheme 5:**
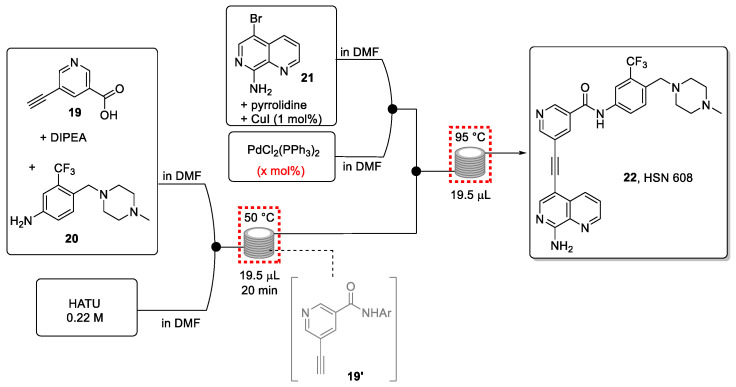
Continuous-flow synthesis of HSN 608.

Thompson et al., therefore, opted to translate the reported batch synthesis into a continuous flow approach (Scheme 5) [66]. This was a worthwhile target to generate sufficient material in support of in vivo experimentation, necessary for the further development of this novel anticancer drug candidate. 

The chosen approach exploits the use of design of experiments (DoE) and high-throughput experimentation (HTE), coupled with DESI-MS as a quick characterization technique. Integrating these enabling techniques facilitated the optimization of the flow process in view of gaining a full understanding of potential side-products geared toward their subsequent minimization.

**Figure 4:**
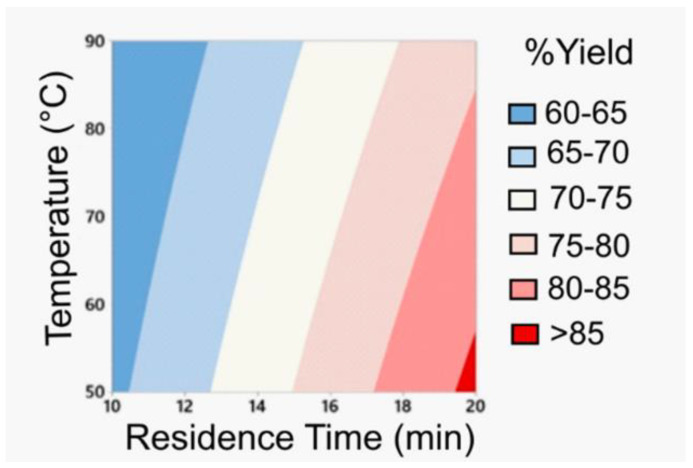
Optimization process for the amidation step exploiting DoE; Reprinted with permission from *Org. Process Res. Dev.* 2020, *24*, 2240–2251. Copyright 2020 American Chemical Society.

The first stage involves a DoE for the amide coupling in flow mode. Based on batch and flow experiments, the most favorable conditions were identified to safely use the unstable and potentially explosive amidation reagent HATU. The information obtained from the DoE optimization process (Figure 4) led to the synthesis of the desired amide intermediate in a very good yield (86%) within a short residence time of only 20 min.

**Figure 5:**
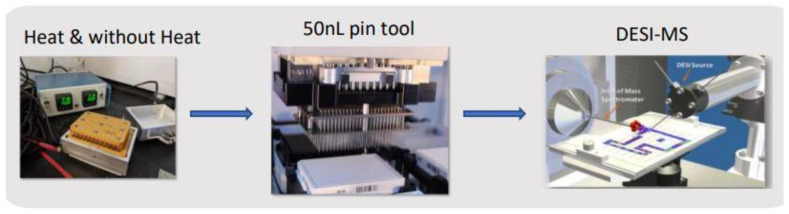
General workflow for the high-throughput DESI-MS based experiments; Reprinted with permission from *Org. Process Res. Dev.* 2020, *24*, 2240–2251. Copyright 2020 American Chemical Society.

Next, suitable conditions for the Sonogashira coupling reaction with aryl bromide **21** were sought using DoE/HTE experimentation coupled with DESI-MS for the instantaneous quantification of the desired reaction product (Figure 5). The experiments were performed in a single DESI-MS plate to produce more than 300 unique data points. This provided information on the optimal choice of base, solvent, and temperature, and additionally helped to identify the rate-limiting step in the production of the final product. 

The two reactions were then successfully telescoped, leading to a fast synthesis of the target drug **22** in good yield (54%). This micro-reactor approach (volume of 20 μL) enabled the generation of almost 100 mg of product in 12 h while consuming very small amounts of reactants during the optimization process. Larger flow reactors are available to provide gram quantities of the drug candidate **22** for further explorations.

### 3.5. Prexasertib

Prexasertib (**28**) is an inhibitor of checkpoint kinase 1 (CHK1), a protein involved in DNA replication and the repair of damaged DNA. Research into the efficacy of prexasertib for the treatment of acute myeloid leukemia, myelodysplastic syndrome, rhabdomyosarcoma, and medulloblastoma is ongoing. Due to the low oral bioavailability, the drug is administered via infusion [67,68,69]. To render the drug more water-soluble, a semi-continuous process was targeted by a team from Eli Lilly to produce the required monolactate salt of **28** [70]. 

**Scheme 6:**
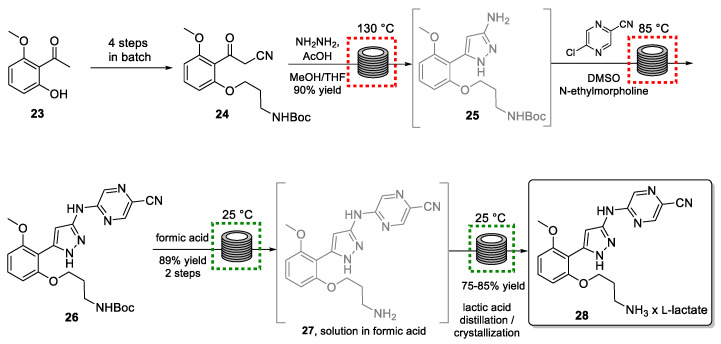
Semi-continuous synthesis of prexasertib monolactate monohydrate (**28**).

The synthetic strategy involves seven steps, of which the last four are performed in continuous flow mode (Scheme 6). Eight continuous unit operations were integrated into this flow process producing three kilograms of the target per day.

Starting from acetophenone **23**, an advanced building block (**24**) was prepared in batch mode. This material was then converted into pyrazole **25** via a hydrazine-mediated high-temperature condensation reaction. This step called for exploiting flow processing in view of the safety concerns associated with the harmful and unstable hydrazine reagent. A further advantage of performing the latter parts of this synthesis within contained flow reactor systems is that only small quantities of chemicals are processed at any given time and that operators are not in contact with intermediates, as well as the final drug where the potency of these cytotoxic agents would pose a substantial risk.

A fundamental aspect of this flow-based strategy was the integration of process analytical technology (PAT) such as HPLC or DSC. Online analysis plays a crucial role in automated flow systems and many reports on the resulting benefits can be found in the literature [71,72,73]. For instance, online HPLC analysis allowed for the detection of a disturbance: after several days of processing, the outflow from the S_N_Ar reaction giving **26** began to exhibit elevated levels of pyrazole precursor **25,** as well as a deprotected derivative which would be in the form of an HCl salt. As this salt would be difficult to be removed in the subsequent crystallization, its generation needed to be avoided at this stage. Therefore, the flow process was interrupted, and the contaminated material was rejected. Investigations led to the conclusion that *N*-ethylmorpholine (used as a base in the S_N_Ar reaction) was slowly evaporating from the feed stream, resulting in a pH imbalance, and therefore leaving some hydrogen chloride from the previous step in the solution. Therefore, had online PAT not been available, the disturbance would likely have gone undetected for a longer period risking the loss of substantial quantities of product.

Product **26** was subsequently subjected to Boc-group removal using formic acid, as well as salt exchange (formate to lactate), as part of the flow process. Final purification of target **28** was achieved via already established batch protocols. By using this integrated continuous process for small volume continuous (SVC) manufacturing, the authors succeeded in producing 24 kg of prexasertib monolactate monohydrate (**28**) for use in clinical trials. 

Additional studies towards automated access to prexasertib and various analogs have been reported recently by Liu et al. outlining the exploitation of solid-phase assisted synthesis for these targets [74].

### 3.6. Merestinib

Merestinib (**35**) is an experimental anticancer drug under development by Eli Lilly. It is studied for the treatment of advanced biliary tract cancer, non-small cell lung cancer, and solid tumors [75,76]. The batch synthesis of the drug presents risks due to known potentially genotoxic impurities (GTIs).

Recently, the Small Molecule Design and Development department within Eli Lilly’s research laboratories published a 20 kg demonstration campaign which describes the drivers for continuous manufacturing (CM) and Small Volume Continuous (SVC) process development for this drug, highlighting various advantages and challenges experienced during the process [77,78].

**Scheme 7:**
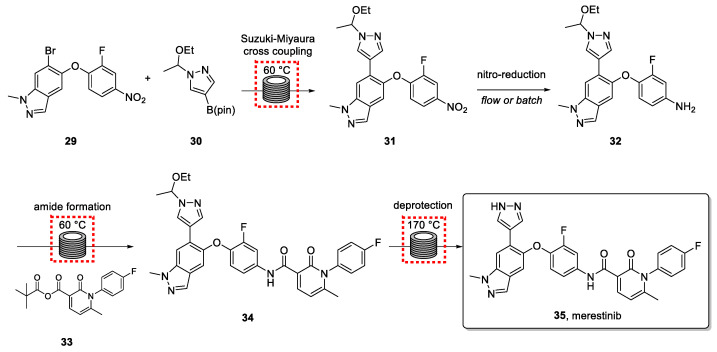
Synthetic route for the synthesis of merestinib via a continuous process.

The synthetic route is shown in Scheme 7. Previous work from Cziesla et al. [79] formed the basis for developing a continuous route that needed to include an effective impurity control strategy.

The first step involves a Suzuki-Miyaura cross coupling between indazole bromide **29** and pyrazole **30**, followed by the hydrogenation of the pending nitro group of intermediate **31**. Subsequent amide bond formation was accomplished in the third step using an anhydride coupling partner. The resulting product (**34**) was subjected to aminal deprotection in a high-temperature flow reactor to generate the drug target. The application of flow technology in the last step, which involved forcing conditions, was crucial and demonstrated to be superior to alternative acid-catalyzed methods. 

The intelligent use of intermittent flow stirred tank reactors, continuous extracting methods, and continuous crystallization tools, enabled the highly efficient continuous production of the final API. Superb control over all steps was achieved with the integration of process analytical technology (PAT), to carefully monitor each reaction throughout the campaign.

**Figure 6:**
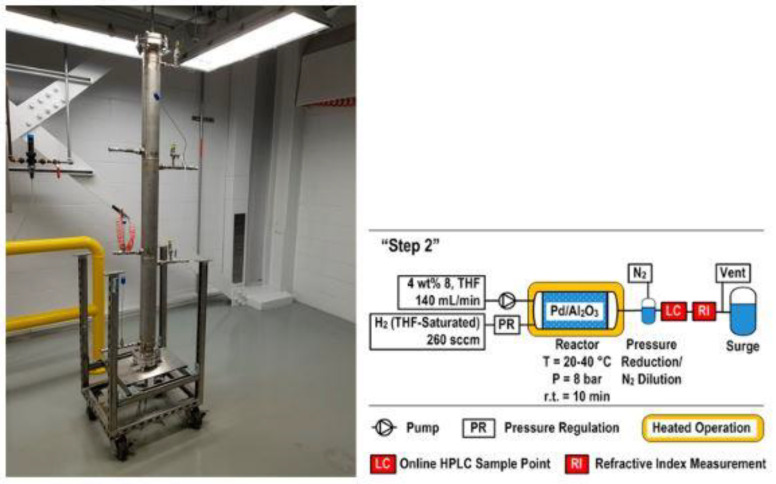
(**Left**) Photograph of the trickle bed reactor developed for the demonstration campaign; (**Right**) schematic representation of continuous nitro-reduction (step 2); Reprinted with permission from *Org. Process Res. Dev.* 2019, *23*, 858–869. Copyright 2019 American Chemical Society.

Although continuous manufacturing (CM) has considerable advantages in industrial drug manufacture, sometimes problems arise from the design and development of the equipment. For example, a trickle bed reactor was designed to conduct the nitro-reduction step in a continuous fashion (Figure 6).

While the productivity and the purity achieved with this trickle bed reactor are impressive (95% yield and >99% purity), reactor fouling problems and low catalyst bed lifetime were challenges observed. Also, the transfer of this technology outside of Eli Lilly was seen as a risk due to the critical nature of reaction monitoring by online HPLC and the operational complexity of the trickle bed reactor. For these reasons, the team decided to abandon the flow-based reduction process in favor of a more traditional batch approach.

Based on learnings from the data collected from this study, Eli Lilly was successful in creating a powerful hybrid batch/CM process affording a total of 183 kg of merestinib.

### 3.7. Capecitabine

Capecitabine (**41**) is a broad-spectrum anticancer agent used for the treatment of metastatic colorectal cancer and as an adjuvant in large-bowel colon cancer and metastatic and advanced breast cancer [80,81]. Being a prodrug, the active compound, 5-fluorocytidine, is released through enzymatic cleavage of the carbamate and inhibits the growth of tumor cells. 

The batch synthesis of capecitabine generally requires long reaction times and significant quantities of Lewis acid (e.g., SnCl_4_). Also, the batch process involves repeated aqueous extractions and other purifications, which is undesirable as it produces copious amounts of waste [82,83].

**Scheme 8:**
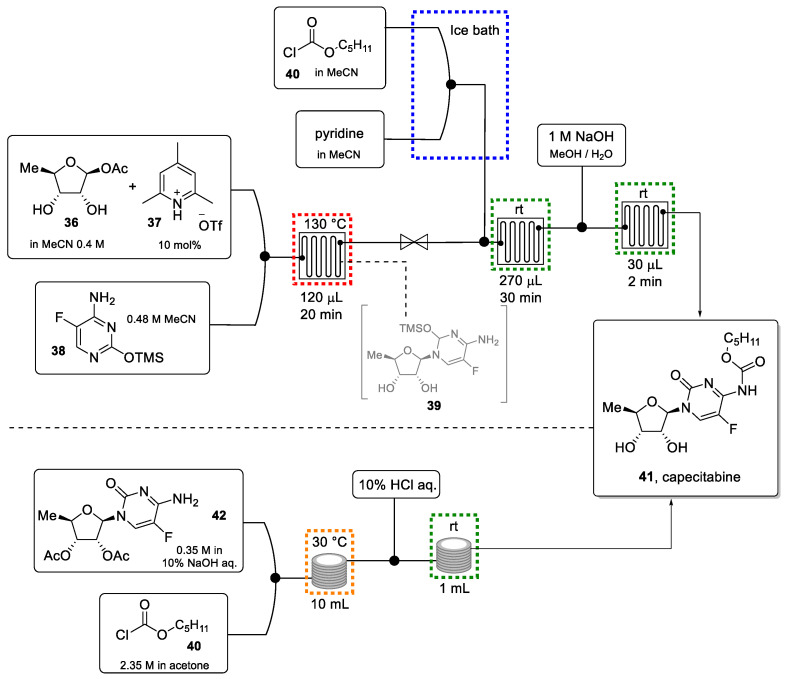
Continuous flow synthesis of capecitabine reported by Jamison (**top**). continuous flow synthesis of the same drug reported by De M. Miranda (**bottom**).

In 2012, Jamison and Shen reported a greener approach that involves a continuous process (Scheme 8) [84]. After initial optimization work, the Vorbrüggen glycosylation reaction between 5-deoxy-ribose (**36**) and the corresponding silylated thymine derivative (**38**) was achieved in only 20 min reaction time using a small PFA tubing microreactor (120 μL volume). The exiting reaction mixture was directed into a second reactor where carbamoylation took place by the introduction of pentyl chloroformate. Acetyl-groups that served as protection groups on the sugar moiety during the initial reactions were eventually removed in a third reactor using a solution of NaOH in MeOH/H_2_O. This three-step flow protocol afforded capecitabine in 72% yield in less than 1 h in a clearly greener and more efficient manner compared to the batch procedure. 

In 2019, De M. Miranda et al. demonstrated a further improvement concerning the carbamoylation/deprotection step towards this anticancer target [85]. Starting from commercially available 2′,3′-diacetoxy-5′-deoxy-5-fluorocytidine (**42**, Scheme 8 bottom) as an advanced intermediate, the carbamate was introduced under Schotten-Baumann conditions thus avoiding the use of acetonitrile and pyridine base. The use of the aqueous base furthermore triggers the deacetylation reaction in this sequence. After optimization, capecitabine was generated in flow with a yield of 81% in a reaction time of only 30 min.

## 4. Essential Building Blocks

As the case studies reported in the preceding section have demonstrated, flow chemistry has been exploited for the improved synthesis of several important anticancer drugs. In these cases, the majority of the synthetic route was conducted as a continuous flow process to benefit from reaction telescoping, allowing the isolation and offline purification of intermediates to be avoided. Academic laboratories, as well as pharmaceutical companies, have thereby explored flow processing to generate small samples of the API as proof-of-concept or to generate multiple kilograms such as in the case of prexasertib and merestinib. While in each case chemists are looking to overcome bottlenecks found in the initial synthesis, it is apparent that the development of more sustainable and safer routes are equally important targets. As the next section will demonstrate, the synthesis of specific building blocks needed in the production of anticancer drugs is another important area that has witnessed the exploitation of flow chemistry in recent years. Here, key drivers for using continuous approaches are improved safety, scalability, and process intensification. Unsurprisingly, the development of a single flow step is typically faster than the realization of a telescoped process, which is attractive for industrial chemists when faced with the decision between process development in batch versus flow mode. 

### 4.1. 4-Fluoro-2-methoxy-5-nitroaniline as a Building Block for Osimertinib

4-fluoro-2-methoxy-5-nitroaniline (**45**) is the core building block of osimertinib (**46**), sold under the brand name Tagrisso^®^. This anticancer drug is an orally active irreversible inhibitor of the epidermal growth factor receptor (EGRF), discovered and developed by AstraZeneca. It is used as a first-line treatment for patients with locally advanced or metastatic non-small cell lung carcinoma (NSCLC) with EGFR sensitizing mutations and as a treatment for patients with EGFR T790M-mutation-positive NSCLC that has progressed on or after EGFR-TKI therapy. The recommended dose in the U.S. is one 80 mg tablet taken once daily [86,87,88]. 

The synthesis of this specific building block is based on the nitration of its precursor, 4-fluoro-2-methoxyaniline (**43**). Nitration reactions are dangerous exothermic transformations due to the presence of strongly oxidizing materials (HNO_3_) and the explosive nature of the reactive intermediates involved. For this reason, nitration reactions are difficult to scale up, generating a bottleneck in the synthesis of the drug [89]. Besides the safety concern, this type of reaction results in particularly challenging batch syntheses because two very important aspects need to be controlled: heat and mass transfer.

**Scheme 9:**
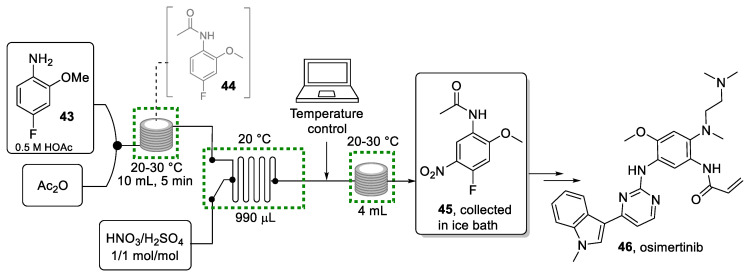
Continuous flow approach for the generation of nitro product **45**.

These considerations sparked research by the Kappe group [90], who subsequently developed safer methodologies for this nitration step in flow mode. The resulting flow process is shown in Scheme 9. The first step involves the acetylation (protection) of the amino group to avoid side reactions during the nitration. Following this, the controlled introduction of the nitro group on the aromatic ring is targeted. 

Initial experiments aimed at a telescoped sequence for lab-scale applications. A solution of substrate **43** dissolved in acetic acid was mixed with a stream of acetic anhydride before entering a PFA reactor coil maintained at ambient temperature. The acetamide intermediate **44** was then combined with the nitration mixture (HNO_3_/H_2_SO_4_) and entered a plate-type microreactor (Ehrfeld FlowPlate Lab Microreactor HC). This microreactor is made from Hastelloy, a nickel-molybdenum alloy particularly resistant to corrosive nitration mixtures. The FlowPlate was maintained at 20 °C using a water-cooled thermostat (Huber CC 304). The reaction mixture was then directed into a reactor coil to allow the reaction to reach completion. The resulting reaction mixture was then collected in an ice/water mixture (1:1 *v*/*v*) in which the product precipitated. To demonstrate the stability of the system, the effluent was collected over a period of 80 min affording product **45** in 82% yield, corresponding to 5.6 g/h, with >99% purity after recrystallization.

**Figure 7:**
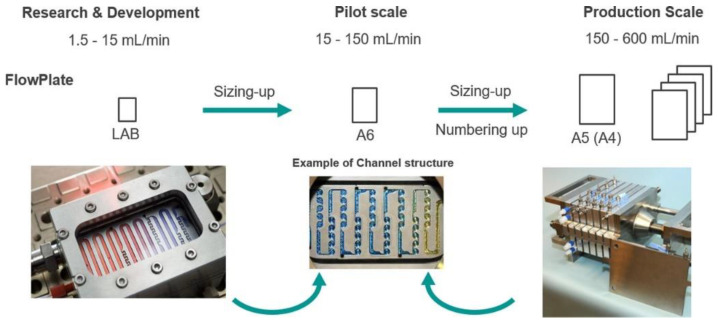
Plate scale-up concept for the nitration process; Reprinted with permission from *Org. Process Res. Dev.* 2020, *24*, 2217–2227. Copyright 2020 American Chemical Society.

To scale up this lab process, the procedure was transferred to Lonza (Visp, Switzerland), using a Modular MicroReaction System (MMRS) equipped with an A5 FlowPlate reactor, which is about four times larger than the original FlowPlate reactor (Figure 7).

Further tuning of the experimental conditions led to the synthesis of the acetylated building block with an impressive throughput of 2 mol/h with >99% purity after filtration and drying. 

### 4.2. (R)-3-[1-(2,6-Dichloro-3-fluorophenyl)ethoxy]-pyridin-2-amine as a Building Block towards Crizotinib

Crizotinib (**49**) is an inhibitor of ATP-competitive multitarget protein kinase and ALK/c-MET/ROS. It is currently considered to be amongst the best treatments for advanced anaplastic lymphoma kinase (ALK)-positive non-small cell lung cancer (NSCLC) [91,92]. The synthesis of the drug requires the reduction of a nitro group (**47**), which was identified as a bottleneck in its synthesis.

**Scheme 10:**
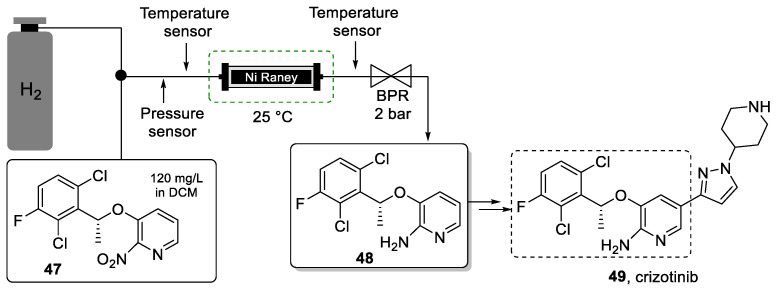
Synthesis of building block **48** using a packed-bed continuous-flow reactor.

Nitro-reduction in halogen-substituted aromatic rings is generally challenging due to competitive side reactions such as debenzylation and dehalogenation. The impurities generated from these reactions can be challenging to remove from the desired reduction product furthermore compounding the need for mild and selective nitro reduction protocols. The use of metal-catalyzed hydrogenation reactions in batch can be cumbersome as the catalyst may be flammable, and its removal by filtration can be tedious. To resolve these issues, Su et al. [93] proposed a continuous flow approach for this step. In this work, the authors show the development of a packed-bed reactor filled with Raney-Ni for the selective nitro-reduction of compound **47**, which is a strategic intermediate in the synthesis of crizotinib (Scheme 10).

After optimization, it was possible to convert 1 kg of starting material **47** within only 6 s residence time in the Ra-Ni reactor. Despite the low substrate concentration (120 mg/L in DCM), this short contact time provided for high throughput, and the low pressure (30 psi) and temperature (25 °C) increased the process safety. The chemoselectivity of this hydrogenation process was very high, avoiding the necessity for challenging downstream separation. In fact, the excellent purity profile of the exiting solution of product **48** meant that it could be used without purification in the next synthetic step. Also, the amount of catalyst used was significantly less when compared to the batch alternative. The immobilization of the catalyst into a readily available HPLC column removed the need for filtrations thus further improving the greenness of the process.

### 4.3. (S)-7-((Tert-butyldiphenylsilyl)oxy)hept-1-yn-4-ol as an Intermediate towards Eribulin

**Figure 8:**
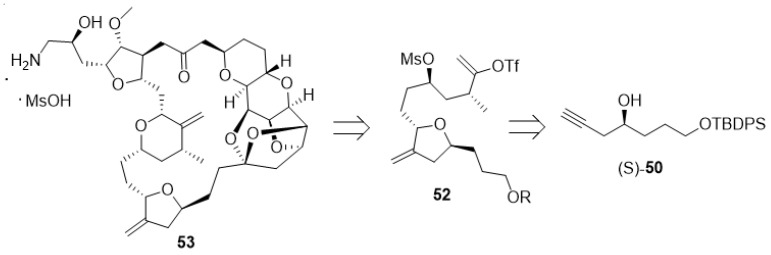
Structure of eribulin mesylate (**53**) and its building blocks **52** and (S)-**50**.

Eribulin mesylate (**53**, Figure 8), currently sold by Eisai under the brand name Halaven^®^, is a synthetic non-taxane microtubule inhibitor used for the treatment of metastatic breast cancer. Anti-microtubule agents attack cells during a certain phase of division and thus are considered cell-cycle specific. Its structure is related to halichondrin B, a complex marine natural product with potent cytotoxic activity [94,95,96,97].

**Scheme 11:**
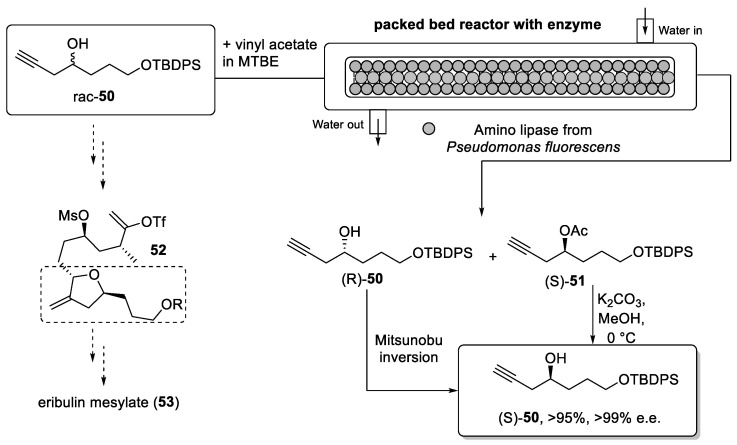
Synthesis of building block (S)-**50** vis flow process using a packed-bed reactor.

Tetrahydrofuran derivative **52** is a key building block for the preparation of eribulin mesylate. This material can be prepared from the enantiomerically pure alcohol (S)-**50** (Scheme 11). The generation of (S)-**50** is a challenging task on scale, as industrial syntheses must avoid toxic and expensive metal catalysts to generate optically pure materials whenever possible. Further issues due to the removal of transition metals to ppm levels must be considered. The separation of racemic products via chiral chromatography is not viable on scale. However, a considerable amount of recent work highlights the possibility to use enzymes for dynamic kinetic resolutions of racemates in a continuous fashion [98,99,100]. 

To provide a greener and more effective strategy for the synthesis of alcohol (S)-**50**, Ghosh et al. [101] developed an enzymatic resolution of the racemic mixture in flow mode. 

The racemic alcohol **50** was prepared in batch mode allowing for the extensive screening of different enzymes. Consequently, a lipase from *P. fluorescens* was selected for further studies because of its high selectivity, robustness, and cost-effectiveness. The use of enzymes in synthesis is generally attractive as this uses mild reaction conditions in aqueous solvent systems. Conducting such transformations in flow mode offers further improvements in relation to better mass transfer. Immobilization of the biocatalyst can circumvent problems associated with the stability of the biocatalyst, loss of enzyme activity as well as enabling simple recovery by filtration. 

Thus, the enzymatic reaction between racemate **50** and vinyl acetate was performed in a packed-bed reactor consisting of two columns (dimensions 250 mm × 4 mm) connected in series. The total weight of the enzyme in the packed bed was 3.6 g and the reactor was used continuously for one week without significant change in productivity. This system provided (S)-**50** as the acetate in excellent yield (96%) and high enantiomeric purity (>99% e.e.). The (R)-enantiomer was thereby interconverted to its enantiomer by a Mitsunobu reaction allowing the use of all the material for the generation of building block **52**. A scale-up experiment was also performed in this report providing 300 g of product through continuous processing. 

The complexity of the chemical structure of eribulin mesylate and the synthetic challenges associated with generating this anticancer drug on scale have also led to further reports concerning the use of flow chemistry. Specifically, researchers from Eisai have disclosed studies for the safe use of DIBAL-H and ^n^BuLi in the synthesis of this target [102]. Here the focus shifted to exploiting the superb heat transfer known for flow processing to use these reagents at higher temperatures than for analogous batch transformations in view of the cost associated with scaled cryogenic reactions.

**Scheme 12:**
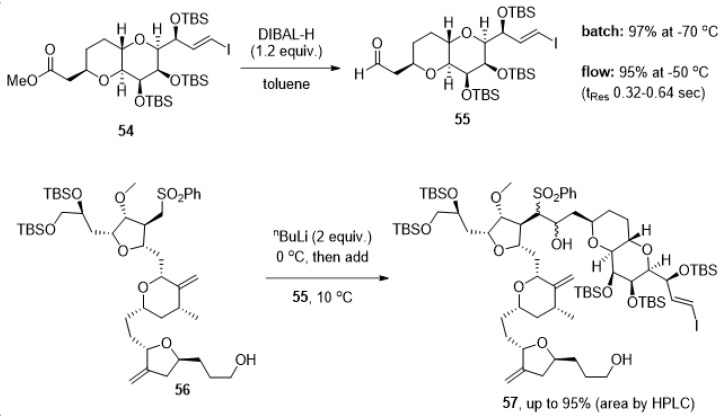
Advantageous use of flow processing in cryogenic reactions towards products **55** and **57**.

As shown in Scheme 12, flow processing allowed to perform the DIBAL-H reduction of ester **54** at temperatures of −50 °C whilst, in batch mode, a temperature of −70 °C was required to minimize the over-reduction of the aldehyde product **55**. Aldehyde **55** was subsequently used in a coupling reaction with sulfone **56** using ^n^BuLi as a base at a low temperature. Using flow processing for this step allowed the use of a temperature of 10 °C (compared to −70 °C in batch) whilst achieving higher conversion to product **57**.

### 4.4. Late Stage Methylation for the Synthesis of AMG 39

AMG 397 (**60**) is a complex macrocyclic structure developed by Amgen currently in phase-1 studies. It has shown promising anticancer activity as an Mcl-1 inhibitor, rendering it usable as a treatment of multiple myeloma (MM) and acute myeloid leukemia (AML) [103,104,105]. Due to the complexity of its structure, the synthesis of this potential drug is challenging, involving more than 40 steps. Late-stage functionalization reactions on such complex structures can be particularly challenging, hence mild and chemoselective transformations play a crucial role in completing the synthesis of the target.

**Scheme 13:**
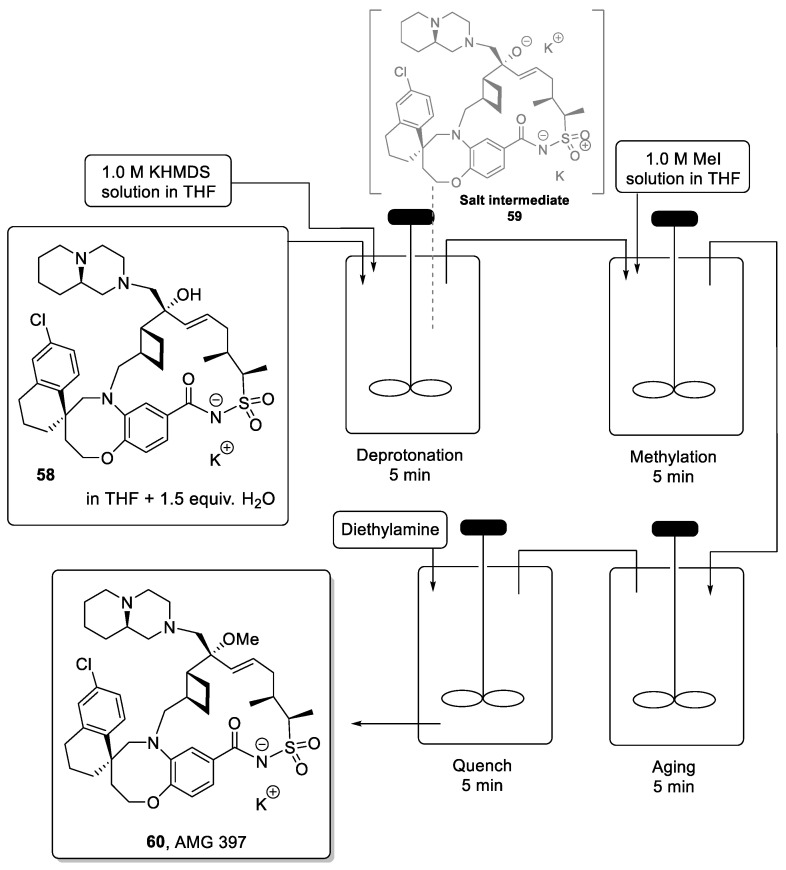
CSTR approach for the methylation step for the synthesis of AMG 397.

However, in the batch campaign, the final methylation step required in the synthesis of AMG 397 led to the over-methylation of the product (i.e., at piperazine moiety) resulting in low isolated yields of the final API along with separation challenges. Moreover, batch-to-batch variability meant that both substrate conversion and product selectivity were inconsistent resulting in a lack of robustness as needed in an industrial process. To overcome these issues, scientists at Amgen developed a flow approach for the selective methylation of the tertiary alcohol precursor (**58**) towards the final API (**60**) (Scheme 13) [106]. 

The optimized procedure utilizes continuous-stirred tank reactors (CSTRs) to achieve deprotonation of the tertiary alcohol as well as the amide present in **58** through the rapid addition of KHDMS as the base. The resulting double salt **59** was then methylated by the addition of MeI. Because salts are involved in this reaction, it was critical to minimize the possibility of precipitation within a standard plug flow reactor. Therefore, CSTRs were connected in series, allowing the processing of 100 g of the substrate without clogging issues. This approach afforded the desired target after isolation in a high yield of 76% and in very high purity. 

The implementation of continuous processes enabled fast and complete deprotonation of substrate **58** without decomposition of the starting material. Precise control over reagent delivery and residence time furthermore mitigated issues of over-methylation and thus enhanced the quality of the entire process. Final recrystallization from aqueous acetic acid and ethanol provided the final API with >98% purity in 75% yield.

## 5. Conclusions

Flow chemistry has played a major part in developing effective syntheses of anticancer drugs. The advantages of miniaturization within flow reactors that lead to improved heat and mass transfer are commonly exploited to gain better process control and selectivity for a variety of transformations. This has been demonstrated for the telescoped flow synthesis of anticancer drugs by academic as well as industrial laboratories to either generate proof-of-concept case studies or for the preparation of multigram quantities of these anticancer drugs and their building blocks in a short time. Scale-up is thereby explored routinely by extending the processing time or applying scale-out principles (i.e., using larger reactors). A recent trend is seen in the exploitation of flow technology for steps that are particularly challenging in the batch route. Pharmaceutical companies thereby opt to target individual steps for flow development, which may be of particular relevance towards the end of the synthesis where the material becomes more valuable, while the reactor containment will mitigate any concerns relating to the cytotoxicity of the final API at the same time. The relevance of natural products towards anticancer drugs has been highlighted in this review, and it is expected that flow technology will continue to facilitate their generation as exemplified in additional studies based on taxol [107], cannabinoids [108], as well as peptide-based systems [109] As this short review has shown, an increase in reported case studies is evident within the last few years demonstrating that flow processing has not only matured as an enabling technology but moreover is accepted and endorsed by both scientists and management within the pharmaceutical industry. A further increase in activity can be expected in this area to realize further autonomy of API production in response to delays and shortages recognized for many drugs during the current pandemic. Continuous flow processing is therefore expected to gain momentum for the supply of these vital medications.

## Figures and Tables

**Table 1:** Overview of anticancer drugs covered.

API Name	API Structure	Cancer Type	Scheme	Ref.
Final API is prepared in flow				
lomustine	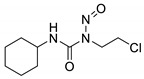	Central nervous system tumors	1	Thompson, D.H., et al. 2019
tamoxifen	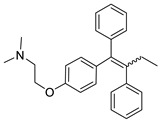	Breast cancer	3	Ley, S.V., et al. 2013
imatinib	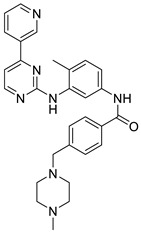	Chronic myelogenous leukemia, gastro-intestinal stromal tumor	4	Jamison, T.F., et al. 2019
HSN 608	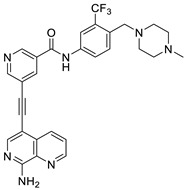	Acute myeloid leukemia	5	Thompso, D.H., et al. 2020
prexasertib	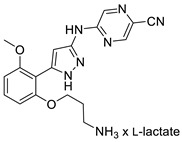	Acute myeloid leukemia (AML), myelodysplastic syndrome, rhabdomyosarcoma, and medulloblastoma	6	Cole, K.P., et al. 2017
merestinib	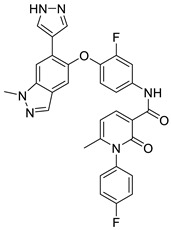	Biliary tract cancer, non-small cell lung cancer and solid tumors	7	Frederick, M.O., et al. 2015
capecitabine	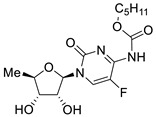	Metastatic colorectal cancer and breast cancer	8	Jamison, T.F., et al. 2012 Miranda, L.S.D.M., et al. 2019
Key precursor to named API is prepared in flow				
osimertinib	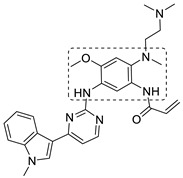	Non-small cell lung carcinoma	9	Hone, C.A., et al. 2020
crizotinib	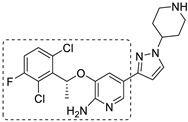	Advanced anaplastic lymphoma kinase (ALK)-positive non-small cell lung cancer (NSCLC)	10	Yu, Z., et al. 2020
Eribulin building blocks	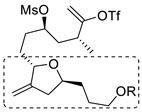	Metastatic breast cancer	11, 12	Kumaraguru, T., et al. 2020 Fukuyama, T., et al. 2016
AMG397	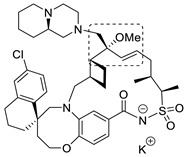	multiple myeloma (MM) and acute myeloid leukemia (AML)	13	Tom, J.K., et al. 2021
